# Childhood Vision Impairment and Refractive Error in Zimbabwe: A Hospital-based Retrospective Study

**DOI:** 10.4314/ejhs.v32i4.8

**Published:** 2022-07

**Authors:** Michael Agyemang Kwarteng, Chido Cleopatra Katsvanga, Samuel Kyei

**Affiliations:** 1 Department of Optometry, Faculty of Science and Engineering, Bindura University of Science Education, Zimbabwe; 2 Discipline of Optometry, College of Health Sciences, University of KwaZulu-Natal, Durban, South Africa; 3 Department of Optometry and Vision Science, School of Allied Health Sciences, College of Health and Allied Sciences, University of Cape Coast, Cape Coast, Ghana

**Keywords:** Vision impairment, childhood, Zimbabwe, refractive error, keratoconus

## Abstract

**Background:**

The objective of this study was to determine the causes and distribution of vision impairment and refractive error among children in Zimbabwe.

**Methods:**

A hospital-based retrospective cross-sectional study was conducted among children (3–16) who attended the Eye Institute, Harare, Zimbabwe, from January 2010 to December 2020. Patients' records were collated, and variables such as visual acuity, ocular morbidities, and vision impairment were analysed.

**Results:**

During this time, 1038 children with mean age of 10.63 ± 3.54 years visited the facility. The majority of them were males (53.2%). Prior to treatment, 9.9% of the children had vision impairment which reduced to 3.5% after intervention. Uncorrected refractive error accounted for the majority of vision impairment (67.0%), followed by keratoconus (7.8%), corneal opacity/ulceration (6.8%), and amblyopia (6.8%), among other conditions. Astigmatism (60.6%) was the most prevalent type of refractive error followed by myopia (37.5%).

**Conclusion:**

The prevalence of childhood vision impairment is higher than that found in similar hospital-based studies conducted in Africa. The most common reason for childhood vision impairment was uncorrected refractive error.

## Introduction

The global estimates of both near and distance vision impairment stands at 2.2 billion of which almost half are preventable and or avoidable (1). It is evident that majority of these cases of vision impairment are due to uncorrected refractive error (1). Even though cases of vision impairment are common among persons aged 50 years and older, it poses a critical developmental challenge when it affects children besides the huge global financial burden of productivity loss of 244 billion United States dollars due to myopia alone (2,3).

The three main domains in which vision impairment influences the cognitive development of children includes its effects on experience, mobility, and self-control in respect to the environment (4). These have consequential effect on children's development, restricting their participation in socio-physical, educational and in later life employment prospects.

Apart from these, early vision impairment among children affect language development as they have limited access to the environment and differential verbal feedback from people within the community (5). This is because vision plays a key role in the perception of objects in totality and context but the visually impaired children depend on sequential observation. This is to say, they see and touch only a part of objects which builds up limited information of an image component (6). The visually impaired have also been found to have high incidence of psychiatry disorders notably anxiety and depressive tendencies (7,8). These undoubtedly have enormous impact on their quality of life given their comparatively extended disability-adjusted life years (9).

This study is the first to focus on vision impairment and refractive error among children in Zimbabwe to ascertain their distinctiveness with respect to vision impairment and refractive error and other associated factors. This is intended to inform the appropriate cohort public health intervention.

## Materials and Methods

The hospital-based retrospective cross-sectional investigation was carried out at the Eye Institute, a private facility in Harare. The facility has the following eye care staff; Ophthalmologists, Optometrists, Ophthalmic nurses, and Opticians. The facility has 10 branches across the country, however, the main branch where this study was conducted has the full complement of the eye health workers, provides surgical services and receives referral from the 9 branches and other care facilities. Paediatric medical records were collected from 2010 to 2020 for children aged 3 to 16 years. The data collection spanned from April to November 2021.

**Inclusion and exclusion criteria**: The study included records of children who visited the facility between January 2010 and December 2020. The data included only information of their maiden medical visit. Medical records that contained insufficient information were excluded.

**Data collection procedure**: A data extraction sheet was used to collect socio-demographic (age, sex, and residence) and clinical profile (presenting visual acuity, best-corrected visual acuity, cause of visual impairment and refractive status).

**Data analysis**: The Statistical Package for Social Sciences (SPSS) version 21 from IBM was used to analyse the data (SPSS Inc, Chicago, IL, USA). The prevalence rate of vision impairment was determined using descriptive statistics for visual acuity ranges, age-specific central tendency measurements, and gender-specific frequency distributions.

**Ethical consideration**: The Research Ethics Committee of the Bindura University of Science Education approved the study protocol (reference number 0004/2021). The study complied with the tenets of the Declaration of Helsinki.

## Results

**Demographics**: There were 1038 (94.9%) out of 1094 folders which met the inclusion criteria and were included in the analysis. Majority were males, 552 (53.2%). The mean age was 10.63 ± 3.54 years with a range of 3 to 16 years. Most (63.7%) of the patients were less than 10 years and majority (82.4%) of them resided inurban centres (Table 1).

**Table 1:** Demographics of patients, Zimbabwe, 2010–2020

Demographics		Sex of patients		Total (%)
			
		Male	Female	
Age groups	< 10	330	331	661 (63.7)
	≥ 10	222	155	377 (36.3)
Location	Urban	461	394	855 (82.4)
	Periurban	91	92	183 (17.6)
Total		552 (53.2)	486 (46.8)	1038 (100)

**Prevalence of vision impairment**: The prevalence of presenting vision impairment in the better eye was 9.9% [95% CI: 8.17 – 11.9] and 3.5% [95% CI: 2.44 – 4.77] after intervention. At presentation, children with mild to moderate vision impairment were 89 (8.6%) as opposed to 28 (2.7%) after intervention. This constitutes 68.5% reduction of vision impairment after intervention. On the other hand, the number of children who presented with blindness reduced from 14 to 8, almost halving the number of children with blindness after the intervention (Table 2).

**Table 1:** Prevalence of vision impairment, Zimbabwe, 2010–2020

	Vision impairment	Sex of patients		Total (%)	P-value
		Female	Male		
1	Normal (0.0 – 0.3 logMAR)	442	493	935 (90.1)	0.761
	Mild (0.32 – 0.5 logMAR)	27	33	60 (5.8)	
	Moderate (0.52 – 1.0 logMAR)	11	18	29 (2.8)	
	Blindness (1.32 – 4.0 logMAR)	6	8	14 (1.3)	
	Normal (0.0 – 0.3 logMAR)	474	528	1002 (96.5)	0.358
2	Mild (0.32 – 0.5 logMAR)	6	15	21 (2.0)	
	Moderate (0.52 – 1.0 logMAR)	3	4	7 (0.7)	
	Blindness (1.32 – 4.0 logMAR)	3	5	8 (0.8)	
	Total	486	552	1038 (100)	

1 = Presenting best visual acuity; 2 = best-corrected visual acuity

**Causes of presenting childhood vision impairment**: The principal cause of childhood vision impairment was uncorrected refractive error (67.0%). Corneal diseases and amblyopia constituted the second tier (21.4%) causes of vision impairment. Cataract, leukocoria, glaucoma and retinopathy contributed marginally (11.6%) to the causes of vision impairment as against refractive error, keratoconus, corneal opacity and amblyopia. However, no female presented with cataract (Table 3).

**Table 2:** Causes of presenting childhood vision impairment, Zimbabwe, 2010–2020

Cause of vision impairment	Sex of patients		Total (%)	P-value
			
	Female	Male		
Refractive error	31	38	69 (67.0)	0.454
Keratoconus	2	6	8 (7.8)	
Corneal opacity/ulceration	2	5	7 (6.8)	
Amblyopia	4	3	7 (6.8)	
Cataract	0	4	4 (3.9)	
Leukocoria	2	1	3 (2.9)	
Retinopathy	2	1	3 (2.9)	
Glaucoma	1	1	2 (1.9)	

Total	44	59	103 (100)	

**Distribution of refractive error**: Astigmatism was the most prevalent (60.6%) type of refractive error followed by myopia (37.5%) of which most (90.9%) of them had low myopia. Hypermetropia constituted the least of the refractive errors, however, their high magnitude could predispose children to amblyopic tendencies (Table 4). The classification of refractive error was based on the study by Naidoo *et al* (10).

**Table 3:** Distribution of Refractive error according to sex, Zimbabwe, 2010–2020

Refractive error (range)	Sex		Total (%)
		
	Female	Male	
Astigmatism (-0.75 to -6.00 D)	83	77	160 (60.6)
Myopia (-0.50 to -9.50 D)	48	51	99 (37.5)
Myopia (-0.50 to -5.75 D)	42	48	90 (90.9)
High myopia (-6.00 to -9.50 D)	6	3	9 (9.1)
Hypermetropia (+2.00 to +14 D)	1	4	5 (1.9)
Moderate (+2.00 to +5.00D)	0	2	2 (40)
High (≥ +5.25 D)	1	2	3 (60)

Total	132	132	264 (100)

Myopia ≥ -0.50 DS, Hyperopia ≥ +2.00 DS, Astigmatism > -0.50 D in isolation or association with hypermetropia or myopia

## Discussion

This hospital-based cross-sectional study determined the prevalence, causes and distribution of vision impairment and refractive error among children attending a private tertiary referral facility in Zimbabwe. Optometrists in Zimbabwe are only found in the private sector; patients would have to rely on private care providers for their refractive error services. The proportion of vision impairment at presentation was reduced more than two folds after intervention (Table 2). This could be due to the fact that uncorrected refractive error which constituted the most cause of the vision impairment is easily treatable with optical aids.

A lower prevalence was recorded for presenting childhood vision impairment in this study compared to a population-based study by Tagoh *et al.* (11), which reported 78.6% as the prevalence for childhood vision impairment. The rural milieu in which Tagoh *et al.* (11) performed their investigation could have played a role in explaining the disparity in the prevalence. In addition, the study's methodology and the availability of subjects may have contributed to its high incidence. Furthermore, people in rural areas of Zimbabwe are more inclined to wait for services provided by government-sponsored and non-governmental organization programs than people in urban areas because of the high expense of accessing health care (12, 13).

This research shows that the prevalence of vision impairment was higher than in similar hospital-based studies in Africa. The prevalence of childhood (5–15 year) vision impairment was 1.3% in Cameroon (14) and 2.5% in South African (15) children (6–18 years). These analyses produced results that differed from the current research results because public eye care facilities for refractive services are readily available and easily accessible in Cameroon and South Africa, and patients would be going to different centers hence making the prevalence lower, whereas this is not the case in Zimbabwe.

Uncorrected refractive error was the most common cause of vision impairment, which is in line with studies in Africa and less developed countries (11, 16, 17). In contrast, in industrialized countries, the new trends are retinal dystrophies and optic neuropathies as the primary causes of childhood vision impairment (18). However, uncorrected refractive error remains the leading global cause of vision impairment and cataract as the main cause of blindness (1). In a study by Penda *et al.* (14), glaucoma was the most common cause of vision loss among Cameroonian children. Disparities between regions may be attributable to difference in access to eye care and preventative measures for childhood ocular disorders, such as refractive services, which could improve with low cost spectacles and ease of accessibility. It is worth noting that keratoconus was the second most common cause of vision impairment in this study, whilst cataracts have been the second most prevalent cause in similar studies (15). Uncertainty abounds as to the reason for this disparity as cataract surgical services are available at the facility. In Africa, however, there is a paucity of knowledge on keratoconus. As stated by Akowuah *et al.* (19) in a systematic review, the prevalence of keratoconus in Africa is 7.9 per cent, with limitations including studies conducted in highrisk populations. Masiwa and Moodley (20) observed in their review study that early diagnosis of keratoconus is critical for prompt intervention and a favourable prognosis, nevertheless, the patients in this study had a relatively high mean age (10.63) at presentation.

The distribution of refractive error among children in urban settlement is skewed towards myopia and astigmatism which is consistent to the findings in this study (10, 21, 22). A major contributing factor might be the active near work among children in upper grades and high schools. Also, the prominent prevalence of keratoconus might have contributed to astigmatism as a leading cause of vision impairment. In the case of noncycloplegic refraction among the children, the prevalence of myopia were likely to be overstated as suggested by Ovenseri-Ogbomo *et al.* (23). Also, myopia was more prevalent in females than males which is consistent with a review study in Africa by Ovenseri-Ogbomo *et al* (23). Girls have a higher risk of myopia than boys due to early developmental differences and less time spent outdoors than boys (24).

In conclusion, the prevalence of childhood vision impairment in urban Zimbabwe is higher than that found in similar hospital-based studies conducted in Africa. The most common reason for childhood vision impairment was uncorrected refractive error. Based on data from this eye hospital, this study gives valuable information about childhood vision impairment in metropolitan Zimbabwe, which will be useful in informing eye care policies. A future population-based study in Zimbabwe will provide a broad description of the country's prevalence and etiology of ocular disorders among children.
